# Neoadjuvant Chemotherapy Approach to Pineal Germinoma: A Case Report

**DOI:** 10.7759/cureus.53325

**Published:** 2024-01-31

**Authors:** Nagham Bazzi, Wajih A Saad, Hala Bazzi, Mohammad Ali Almokdad, Abdo Mghames

**Affiliations:** 1 Medicine, Lebanese University, Beirut, LBN; 2 Oncology, Lebanese University Faculty of Medicine, Beirut, LBN; 3 Dentistry, Saint Joseph University, Beirut, LBN; 4 Medicine, University of Balamand, Beirut, LBN; 5 Medicine, American University of Beirut Medical Center, Beirut, LBN

**Keywords:** neoplasms, germinoma, headache, pineal gland tumor, brain tumor

## Abstract

Intracranial germ cell tumors (GCTs) are rare malignant tumors with a peak incidence around puberty. The pineal region is the most commonly involved area of all intracranial GCTs. Due to the heterogeneous tumor origin, subtypes, and presentation, diagnosis and management are challenging. Complicated pineal germinomas are rarely reported in the literature. Here, we report a rare case of pineal germinoma with hydrocephalus and discuss the potential treatment approach. A 20-year-old boy presented to the hospital with vomiting and a decreased level of consciousness. The brain magnetic resonance imaging (MRI) revealed a pineal tumor. A ventriculoperitoneal shunt was placed to relieve the increased intracranial pressure. The patient underwent a suboccipital craniotomy with excisional biopsy of the pineal region tumor due to its critical location, as imaging studies alone may not be sufficient to establish a definitive diagnosis. Although there has been a rise in reported cases of germinoma tumors, there is currently no standardized therapeutic approach for treating them. Therefore, more randomized controlled cohort studies are necessary to evaluate potential treatments and develop a therapeutic approach.

## Introduction

Intracranial germ cell tumors (GCTs) are rare malignant tumors (except for mature teratomas), accounting for only 0.3-0.5% of all primary intracranial neoplasms with a challenging management and diagnosis [1-3]. Germinomas are the most common germ cell tumors with an incidence of 5 to 1.0/million/year in Western countries and 1.7 to 2.7/million/year in Asian countries [4]. The peak incidence of germinoma occurs in the second decade of life, around puberty, with a male predominance [3,4]. These tumors commonly involve the midline area including the sellar, pineal, and basal regions [4]. The pineal region is the most commonly involved area and accounts for 33-63% of all intracranial GCTs [2,3].

Intracranial germinoma may be presented with growth disturbances, precocious puberty, and hypopituitarism. It also depends on the site of origin of the tumors [4]. Due to the heterogeneous tumor origin, subtypes, and presentation, the diagnosis and management are challenging. However, the international consensus for management is to maintain excellent overall survival with minimal treatment burden with excellent treatment outcomes reported in the literature [1]. Complicated pineal germinomas are rarely reported in the literature and present a rare case of pineal germinoma with hydrocephalus. We also discuss all the salient points of this case and provide a potential treatment approach. Our case was reported in line with the SCARE (Surgical CAse REport) criteria [5]. Ethical consent was given for reporting this case.

## Case presentation

**Figure 1:**
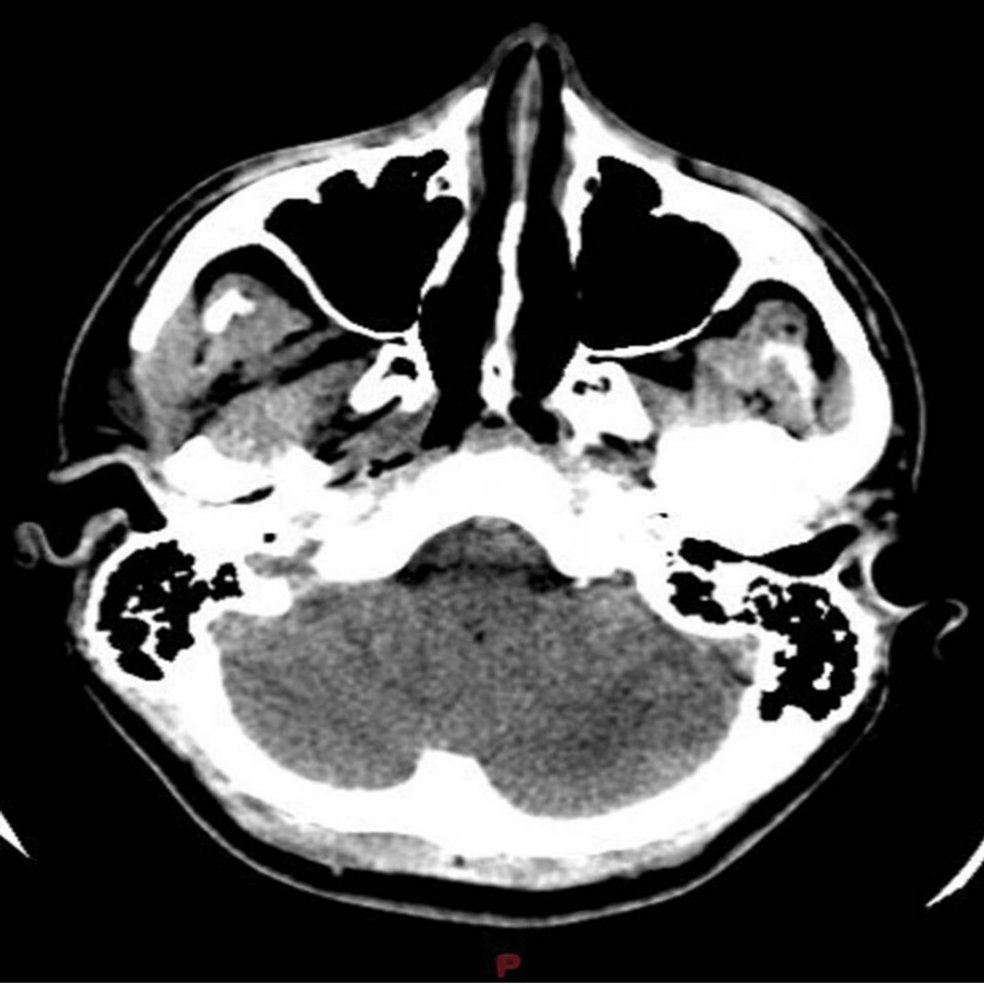
Brain CT scan at presentation

A 20-year-old, previously healthy boy presented to our hospital with a history of severe headaches and vertigo, along with vomiting, severe neck pain with blurry vision, and decreased level of consciousness of a three-day duration. After a complete systemic examination, no physical or neurological abnormalities were reported. A blood workup showed mild leukocytosis (white count = 17900). A cranial computed tomography (CT) showed a soft tissue pineal mass with central calcifications compressing the Sylvian aqueduct, causing upstream hydrocephalus (Figure 1). The brain magnetic resonance imaging (MRI) revealed a tumor in the pineal gland sized 4 cm x 3.1 cm x 3 cm with contrast enhancement, central calcification, and peripheral edema. The tumor was compressing the surrounding structures and mainly the tectal plate, which led to severe compression of the aqueduct of Sylvius. Notably, hydrocephalus with periventricular edema and mild descent of the cerebellar tonsils below the foramen magnum was detected. Unfortunately, the tumor marker level was not available. A whole spinal MRI was also performed with unremarkable findings. Subsequently, a ventriculoperitoneal shunt was placed to relieve the increased intracranial pressure. A cerebrospinal fluid specimen confirmed the presence of mild benign inflammatory cells along with red blood cells without malignant cells. Ten days later, the patient underwent a suboccipital craniotomy under general anesthesia with an excisional biopsy of the pineal region tumor due to its critical location and its radiosensitivity. The postoperative course was smooth without any complications and the patient was discharged home five days later.

**Figure 2:**
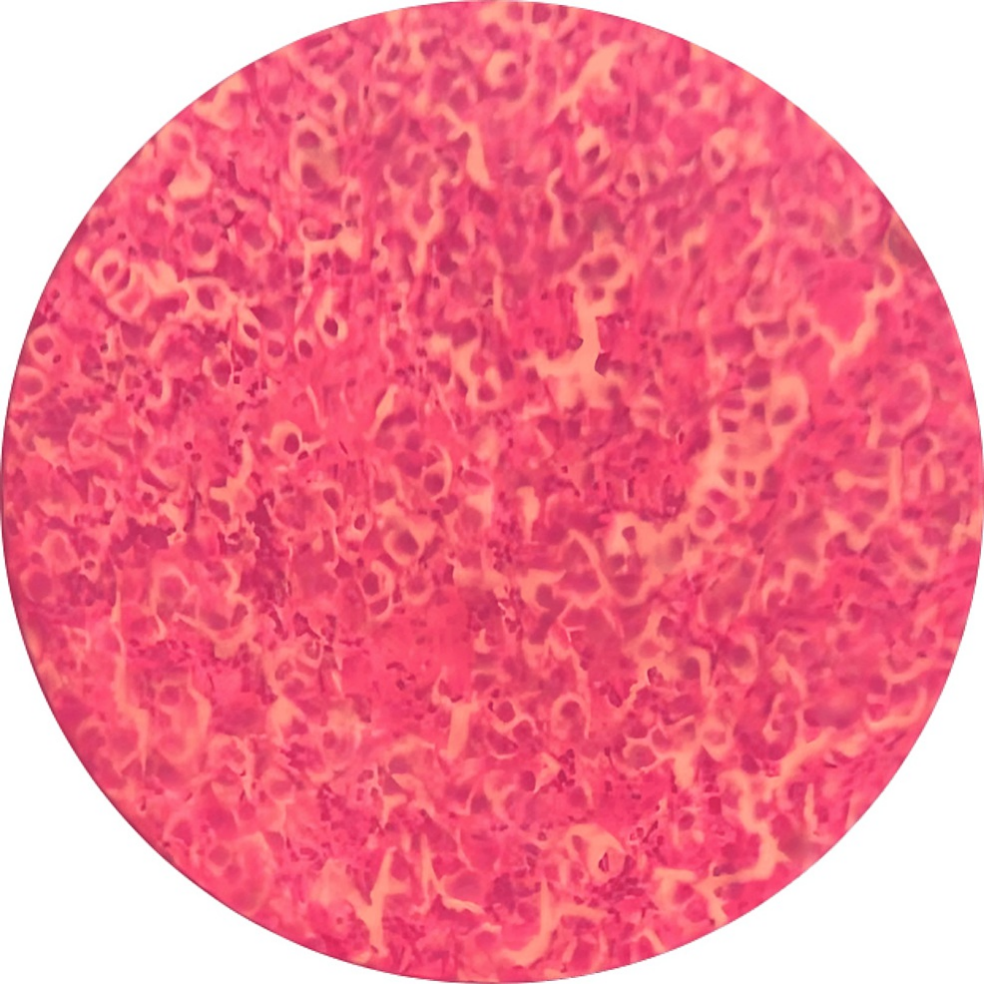
Sheets of large epithelial cells with clearing nuclei and prominent nucleoli at 40X magnification

The histopathological examination revealed large epithelial cells with clearing of nuclei, prominent nucleoli, abundant cytoplasm, and mononuclear infiltrate (Figure 2). The immunohistochemistry showed positivity for OCT-4 and CD117. The diagnosis of germinoma was confirmed. At a follow-up of one month post-craniotomy, the patient was doing fine clinically and tumor size decreased to 3.5 cm x 3cm x 2.5 cm. The VP shunt was still in place. A chemotherapy monthly regimen for four months was started and consisting of etoposide (180 mg IV in 250 cc normal saline solution over three hours), cisplatin (63 mg IV in 500 cc normal saline solution continuous over 20 hours), vincristine (2 mg IV in 50 cc normal saline solution over 10 minutes) and cyclophosphamide (2100 mg IV in 250 cc normal saline solution over 20 hours). The patient has started recently radiotherapy sessions and was satisfied with the treatment received.

## Discussion

Central nervous system GCTs are uncommon intracranial tumors that share common features with ovarian and testicular germ cell tumors. The pathophysiology of extragonadal germ cell cancers is not fully elucidated but could be attributed to one of these two hypotheses: embryonal germ cells could have migrated abnormally along the urogenital ridge, which contributed to their dissemination to wrong places and thereby to tumor growth, and the alternative one says that germ cells may first migrate to the gonads before spreading in reverse to other sites [6].

Clinical presentation of GCT including germinomas might differ according to their location in the central nervous system [7]. Patients may present with symptoms of obstructive hydrocephalus, including headaches, loss of upward gaze, nausea, vomiting, and dyskinesia, if the tumor is located in the pineal region, whereas tumors that arise in the suprasellar region might present with diabetes insipidus or any hormone deficiency by compressing the hypothalamus or the pituitary gland [6,7]. The presence of a hormonal dysregulation hinders the diagnosis whereas symptoms of obstructive hydrocephalus steer the differential diagnosis into a neurological abnormality, which explains the delayed diagnosis in the Davoudi et al. study and the early diagnosis in our case [7]. This emphasizes the need for a greater level of awareness of various presentations that fall under the diagnosis of germinomas.

The diagnosis of germinomas is challenging and requires an in-depth assessment of hormonal levels in order to exclude other potential causes. Imaging examination of a germinoma might show a blurred border, high-density uniformly enhanced tumor on a CT scan. In contrast, T1, T2, and enhanced MRIs show different characteristics of these tumors and thereby are considered standard imaging for germ cell tumors [6].

Patients diagnosed with germinomas located in unusual sites, including basal ganglia, cerebellum, or brainstem are associated with a worse prognosis when compared to patients diagnosed with germinomas located in the pituitary or pineal glands [8]. In addition, pineal germinomas are usually associated with a good prognosis, showing survival at a median of 7.25 years from the end of treatment [9]. Treatment protocols are still unclear in germ cell tumors. Patients with localized germinomas might benefit from a chemotherapy regimen followed by the irradiation of the whole ventricle, in contrast to patients diagnosed with metastatic germinomas who should undergo craniospinal irradiation [10].

For localized germinomas, the recommended treatment is cranial radiation therapy (CRT) with a specific dose, followed by a boost dose. Chemotherapy is not recommended unless CRT is not possible, in which case carboplatin and etoposide may be used. However, CRT can cause long-term side effects, so reduced-dose CRT, focal radiation therapy, and chemotherapy-only regimens are being studied as alternatives. Treatment should be tailored to each patient and tumor, and further research is needed to determine the best approach that balances effectiveness and safety [10,11].

## Conclusions

Intracranial germ cell tumors, particularly germinomas, present a formidable challenge in diagnosis and management due to their rarity, diverse presentation, and critical locations within the brain. The varied clinical manifestations depending on tumor location highlight the importance of vigilance toward diverse presentations, emphasizing the need for a high index of suspicion and thorough hormonal evaluations to differentiate from other pathologies. The reported case demonstrates successful management involving surgical intervention, followed by a tailored chemotherapy regimen and ongoing radiotherapy. While this protocol yielded positive outcomes in this instance, the lack of a universally accepted therapeutic approach underscores the imperative for further randomized controlled cohort studies to establish more definitive and standardized treatment guidelines.
